# Menopause and colorectal cancer

**DOI:** 10.1054/bjoc.1999.1084

**Published:** 2000-06

**Authors:** S Franceschi, S Gallus, R Talamini, A Tavani, E Negri, C La Vecchia

**Affiliations:** Servizio di Epidemiologia, Centro di Riferimento Oncologico, via Pedemontana, Aviano (PN), 33081, Italy; Istituto di Ricerche Farmacologiche ‘Mario Negri’, via Eritrea 62, Milano, 20157, Italy; Istituto di Statistica Medica e Biometria, Università degli Studi di Milano, via Venezian 1, Milan, 20133, Italy

**Keywords:** colon cancer, rectal cancer, menopause, hormone replacement therapy, weight, case–control study

## Abstract

Post-menopausal women who have never used hormone replacement therapy have a higher risk of colon, but not rectal, cancer than do premenopausal women of the same age, socio-cultural class and dietary habits. Such risk increase seems to last about 10 years and to be restricted to lean women, a group who have lower levels of oestradiol after ovarian function ceases after menopause. © 2000 Cancer Research Campaign


*British Journal of Cancer* (2000) **82**(11), 1860–1862
© 2000 Cancer Research Campaign
doi: 10.1054/ bjoc.1999.1084, available online at http://www.idealibrary.com on IDEAL®

### Menopause and colorectal cancer

**S Franceschi[1], S Gallus[2], R Talamini[1], A Tavani[2], E Negri[2] and C La Vecchia[2,3]**

[1]Servizio di Epidemiologia, Centro di Riferimento Oncologico, via Pedemontana, 33081 Aviano (PN), Italy; [2]Istituto di Ricerche Farmacologiche 'Mario Negri', via Eritrea 62, 20157 Milano, Italy; [3]Istituto di Statistica Medica e Biometria, Università degli Studi di Milano, via Venezian 1, 20133 Milan, Italy.

**Summary** Post-menopausal women who have never used hormone replacement therapy have a higher risk of colon, but not rectal, cancer than do premenopausal women of the same age, socio-cultural class and dietary habits. Such risk increase seems to last about 10 years and to be restricted to lean women, a group who have lower levels of oestradiol after ovarian function ceases after menopause. © 2000 Cancer Research Campaign

**Keywords:** colon cancer; rectal cancer; menopause; hormone replacement therapy; weight; case–control study

Hormone replacement therapy (HRT) use has been found to be inversely associated with colorectal cancer in several case–control and cohort studies (Franceschi and La Vecchia, 1998). A meta-analysis of 20 studies published up to 1997 on colon cancer in women led to a summary relative risk (RR) of 0.85 (95% confidence interval (CI) 0.73–0.99) (Hebert-Croteau, 1998). The suggested protective effect of HRT was stronger in studies published since 1990 and among current or recent users (RR 0.69; 95% CI 0.52–0.91).

The adverse effect of HRT use in respect to cancer of the breast, endometrium (IARC, 1999) and, possibly, ovary (Negri et al, 1999) is consistent with the well established direct association between age at menopause and risk at these sites. For breast cancer, the increase in risk for each year of HRT use is similar to the effect of 1-year delay in menopause (Collaborative Group on Hormonal Factors in Breast Cancer, 1997). For colorectal cancer, no consistent relationship has been shown with age at menopause (La Vecchia and Franceshi, 1991; Jacobs et al, 1994; Kampman et al, 1994, 1997; Martinez et al, 1997; Troisi et al, 1997), but the issue has not been considered in depth. In order to further clarify the influence of menopause on colorectal cancer, we have re-evaluated data collected in Italy between 1985 and 1996.

### SUBJECTS AND METHODS

The data were derived from two case–control studies on colorectal cancer: the first conducted between 1985 and 1991 in the greater Milan area (Negri et al, 1989) and the province of Pordenone, in Northeast Italy (Franceschi et al, 1991); and the second conducted between 1992 and 1996 in the provinces of Pordenone and Gorizia in Northeast Italy; the urban areas of Milan and Genoa and the province of Forlì in northern Italy; Latina in central Italy; and the urban area of Naples in southern Italy (Talamini et al, 1998). Centrally trained interviewers identified and questioned cases of colorectal cancer and controls during their hospital stay.

Cases included 1536 women below age 75 years with histologically confirmed cancer (i.e. diagnosed within the year before interview) of the colon (994 cases, median age 61) and rectum (542 cases, median age 62), admitted to the major teaching and general hospitals in the areas under surveillance.

The control group included 3110 women younger than 75 years (median age of 57), residing in the same geographical areas and admitted for a wide spectrum of acute, non-neoplastic, non-digestive tract, non-hormone-related disorders to the same network of hospitals from which cases were recruited. Of these, 43% were admitted for traumas, 14% had non-traumatic orthopaedic disorders, 13% had acute surgical conditions, 6% had eye conditions and 24% had other miscellaneous diseases, such as ear, nose and throat, skin, or dental disorders. Cases and controls had the same referral pattern. Fewer than 5% of cancer cases and control subjects refused to be interviewed.

Structured questionnaires were used to collect information on sociodemographic factors, personal characteristics and habits (such as smoking, alcohol and coffee consumption), frequency of consumption of selected foods and a problem-oriented medical history. Information was also collected on menstrual and reproductive factors and on the use of oral contraceptives and HRT (Fernandez et al, 1998). Women were considered post-menopausal if they reported no menstrual periods in the 12 months prior to colorectal cancer diagnosis or hospital admission (controls), or had undergone hysterectomy and/or bilateral oophorectomy.

To examine the effect of menopause on colorectal cancer risk independently of the effect of HRT, most analyses were restricted to women who had never used HRT. Odds ratios (ORs), together with the corresponding 95% CIs, were derived from unconditional multiple logistic regression equations, fitted by the method of maximum likelihood (Breslow and Day, 1980). Terms included in the regression equations were age (in quinquennia plus a continuous term), centre, study period, education, parity, vegetable intake and body mass index (BMI, kg $m^{-2}$). Allowance for other potentially confounding variables (smoking habit, alcohol drinking, coffee intake, meal frequency, family history of colorectal cancer and, for the more recent study, physical activity, total energy intake and aspirin use) did not substantially modify any of the ORs in Tables 1 and 2.

*Received 14 July 1999*
*Revised 1 October 1999*
*Accepted 1 October 1999*

*Correspondence to:* S Franceschi

**Table 1** Odds ratios (ORs) and corresponding 95% confidence intervals (CIs)[a] of cancer of the colon and rectum according to menopausal status, type of and age at menopause, and years since menopause. Italy, 1985–1996

| | Colon cancer | | Rectal cancer | | Controls |
|---|---|---|---|---|---|
| | **No.** | **OR (95% CI)** | **No.** | **OR (95% CI)** | **No.** |
| Menopausal status[c,d] | | | | | |
| Pre- | 170 | 1[b] | 88 | 1[b] | 897 |
| Post | 770 | 1.46 (1.05–2.04) | 429 | 1.19 (0.76–1.86) | 1973 |
| HRT-users | 54 | 0.93 (0.60–1.43) | 23 | 0.56 (0.31–1.03) | 235 |
| Type of menopause[c,d] | | | | | |
| Natural | 619 | 1[b] | 343 | 1[b] | 1578 |
| Surgical | 149 | 1.10 (0.88–1.38) | 85 | 1.19 (0.91–1.57) | 390 |
| Age at menopause (years)[c,d] | | | | | |
| <45 | 124 | 1[b] | 84 | 1[b] | 390 |
| 45–49 | 199 | 1.21 (0.92–1.58) | 113 | 0.96 (0.69–1.32) | 534 |
| 50–52 | 288 | 1.46 (1.12–1.89) | 152 | 1.05 (0.77–1.42) | 630 |
| ≥53 | 157 | 1.17 (0.87–1.57) | 78 | 0.76 (0.53–1.08) | 412 |
| $\chi_1^2$(trend) | | 2.20; *P*=0.14 | | 1.33; *P*=0.25 | |
| Years since menopause[c,d] | | | | | |
| Pre-menopausal | 170 | 1[b] | 88 | 1[b] | 897 |
| <10 | 254 | 1.66 (1.21–2.28) | 120 | 1.20 (0.79–1.82) | 698 |
| 10–19 | 309 | 1.18 (0.80–1.73) | 191 | 1.26 (0.78–2.06) | 851 |
| ≥20 | 205 | 1.09 (0.69–1.72) | 116 | 1.14 (0.64–2.01) | 417 |
| $\chi_1^2$(trend)[e] | | 5.07; *P*=0.02 | | 0.06; *P*=0.80 | |

[a]Estimates from unconditional multiple regression equations including terms for age, study centre, educational years, parity, vegetable intake and body mass index. [b] Reference category. [c] Ever users of hormone replacement therapy (HRT) are not included. [d] Sums do not add up to the total because of a few missing values. [e] Premenopausal women are excluded from trend analyses.

**Table 2** Odds ratios (ORs) and corresponding 95% confidence intervals (CIs)[a,b,c] of colon cancer by years since menopause and body mass index (BMI); Italy, 1985–96

| | Body mass index (BMI) | | | | | |
|---|---|---|---|---|---|---|
| **Years since menopause**[b] | **<23** | | **23–26** | | **>26** | |
| | **Cases:Controls**[c] | **OR (95% CI)** | **Cases:Controls** | **OR (95% CI)** | **Cases:Controls** | **OR (95% CI)** |
| Premenopausal | 85:392 | 1[d] | 45:250 | 1[d] | 40:255 | 1[d] |
| <10 | 95:184 | 2.21 (1.33–3.69) | 82:230 | 1.37 (0.77–2.45) | 77:281 | 1.34 (0.75–2.40) |
| 10–19 | 104:244 | 1.27 (0.67–2.40) | 89:264 | 0.77 (0.37–1.61) | 116:341 | 1.34 (0.69–2.61) |
| ≥20 | 71:137 | 0.97 (0.45–2.08) | 66:122 | 0.85 (0.36–1.99) | 68:157 | 1.24 (0.57–2.70) |
| $\chi_1^2$(trend)[e] | | 6.00; *P*=0.02 | | 1.37; *P*=0.25 | | 0.16; *P*=0.69 |

[a]Estimates from unconditional multiple regression equations including terms for age, study centre, educational years, parity and vegetable intake. [b]Ever users of hormone replacement therapy are not included. [c]Sums do not add up to the total because of missing values. [d]Reference category.
[e]Premenopausal women are excluded from trend analyses.

### RESULTS

Table 1 shows the distribution of cases of cancer of the colon and rectum and control subjects according to menopause-related variables. Compared to premenopausal, post-menopausal women who had never used HRT were at 1.5-fold increased risk (95% CI 1.1–2.0) of colon cancer. Conversely, women who had ever taken HRT had an OR of 0.9 (95% CI 0.6–1.4). Type of menopause did not seem to modify colon cancer risk. Women who reported menopause at age 50–52 years had an OR of 1.5 (95% CI 1.1–1.9) compared to those whose menopause was at age 44 or less, but the trend in risk was not linear. Women whose menopause occurred 1–9 years before diagnosis had a substantially higher risk (OR = 1.7, 95% CI 1.2–2.3) than premenopausal ones. Thereafter, ORs declined progressively with time since menopause. Trend by years since menopause among post-menopausal women was significant (Table 1). For rectal cancer, no significant associations with age at menopause or years since menopause were found. Ever users of HRT showed decreased risk of rectal cancer compared to premenopausal women (Table 1).

The relation of colon cancer risk with time since menopause differed significantly according to BMI tertile (Table 2). The risk elevation in the 10 years subsequent to menopause seemed restricted to women with BMI below 23 (OR = 2.2; 95% CI 1.3–3.7) ($\chi^2_1$ for trend = 6.00, $P$ = 0.02). Conversely, the decline in risk by years since menopause was not significant in women whose BMI was between 23 and 26 ($\chi^2_1$ for trend = 1.37, $P$ = 0.24) and was absent among those with BMI higher than 26 ($\chi^2_1$ for trend = 0.16, $P$ = 0.69). Trends by age at and years since menopause did not differ between women with natural and those with surgical menopause.

*© 2000 Cancer Research Campaign* 

### DISCUSSION

The main finding is that post-menopausal women have a higher risk of colon, but not rectal, cancer than do premenopausal women. An adverse effect of late menopause is consistent with the findings of some previous studies (Kampman et al, 1994; Martinez et al, 1997). In addition, colon cancer incidence data in developed countries show an excess in males, compared to females, from approximately age 50 years (Parkin et al, 1997). Relatively greater colon cancer incidence in younger women than men suggests that oestrogen exposure may be predisposing. However, in the form of HRT, oestrogens seem to be protective (Franceschi and La Vecchia, 1998). In fact, HRT users in our study were not at increased risk of colon cancer compared to premenopausal women. Furthermore, the risk increase after menopause seems transient and restricted to lean women, i.e. those who experience lowest levels of circulating oestrogens and sex-hormone binding globulins when ovarian function ceases (Burger et al, 1995).

Risk increase in the years subsequent to menopause, however, may be due to endocrinological changes other than the fall in oestrogens, such as high levels of follicle-stimulating hormone, lutenizing hormone, or other pituitary tropic hormones (e.g. growth-hormone) in the peri- and early post-menopausal period (Burger et al, 1995). Additional complexity is added by the discovery of a second class of oestrogen receptors (ER) (i.e. ER-β) which has a different tissue distribution from the classical ER-α, as well as a different response after binding ligand (Kuiper and Gustafson, 1997). Finally, colonic mucosa can regulate oestrogen action by locally expressed metabolizing enzymes (English et al, 1999). Interconversion of oestradiol ($E_2$) to less potent oestrone ($E_1$) was lower in colonic tumours compared to normal mucosa (English et al, 1999). $E_1$, but not $E_2$, was shown to significantly decrease proliferation when added to colonic epithelial cell lines (Di Domenico et al, 1996; English et al, 1999). Gradually after menopause, the ratio between $E_1$ and $E_2$ increases, reflecting the declining follicular steroidogenesis and the increasing importance of peripheral aromatization of androgens to $E_1$ (Rannevik et al, 1995). $E_1$, but not $E_2$, is contained in one of the most commonly prescribed HRT (i.e. conjugated oestrogens) (English et al, 1999).

ORs by age at and recency of menopause were unmodified by stratification and adjustment for single year of age. This ensures that age did not account for our findings. Infrequent HRT use in Italy allowed the study of menopause in a large number of women who had never used such therapy. Misclassification of self-reported age at natural menopause does not seem systematic (Hahnn et al, 1997), and our findings among women who underwent natural menopause were replicated among those who underwent surgical menopause. However, the potential influence of menopause on colon cancer should be reconsidered in larger studies or pooled analyses to evaluate risk modification by single year since menopause.

### ACKNOWLEDGEMENTS

This work was conducted with the contribution of the Italian Association for Research on Cancer, Milan, Italy. We wish to thank Mrs L Mei for editorial assistance.

### REFERENCES

Breslow NE and Day NE (1980) *Statistical Methods in Cancer, Vol. I. The Analysis of Case–control Studies*. IARC Scientific Publication No 32. International Agency for Research on Cancer: Lyon

Burger HG, Dudley EC, Hpper JL, Shelley JM, Green A, Smith A, Dennerstein L and Morse C (1995) The endocrinology of the menopausal transition: a cross-sectional study of a population-based sample. *J Clin Endocrinol Metab* **80**: 3537–3545

Collaborative Group on Hormonal Factors in Breast Cancer (1997) Breast cancer and hormone replacement therapy: collaborative re-analysis of data from 51 epidemiological studies of 52 705 women with breast cancer and 108 411 women without breast cancer. *Lancet* **350**: 1047–1059

Di Domenico M, Castoria G, Bilancio A, Migliaccio A and Auricchio F (1996) Estradiol activation of human colon carcinoma-derived Caco-2 cell growth. *Cancer Res* **56**: 4516–4521

English MA, Kane KF, Cruickshank N, Langman MJS, Stewart PM and Hewison M (1999) Loss of estrogen inactivation in colonic cancer. *J Clin Endocrinol Metab* **84**: 2080–2085

Fernandez E, La Vecchia C, Braga C, Talamini R, Negri E, Parazzini F and Franceschi S (1998) Hormone replacement therapy and risk of colon and rectal cancer. *Cancer Epidemiol Biomarkers Prev* **7**: 329–333

Franceschi S and La Vecchia C (1998) Colorectal cancer and hormone replacement therapy: an unexpected finding. *Eur J Cancer Prev* **7**: 427–438

Franceschi S, Bidoli E, Talamini R, Barra S and La Vecchia C (1991) Colorectal cancer in Northeast Italy: reproductive, menstrual and female hormone-related factors. *Eur J Cancer* **27**: 604–608

Hahnn RA, Eaker E and Rolka H (1997) Reliability of reported age at menopause. *Am J Epidemiol* **146**: 771–775

Hébert-Croteau N (1998) A meta-analysis of hormone replacement therapy and colon cancer in women. *Cancer Epidemiol Biormarkers Prev* **7**: 653–659

IARC (1999) *IARC Monographs on the Evaluation of Carcinogenic Risks to Humans, Vol. 72. Some Hormones, Postmenopausal Hormone Therapy, and Hormonal Contraception*. International Agency for Research on Cancer: Lyon

Jacobs EJ, White E and Weiss NS (1994) Exogenous hormones, reproductive history, and colon cancer (Seattle, Washington, USA). *Cancer Causes Control* **5**: 359–366

Kampman E, Bijl AJ, Kok C and van't Veer P (1994) Reproductive and hormonal factors in male and female colon cancer. *Eur J Cancer Prev* **3**: 329–336

Kampman E, Potter JD, Slattery ML, Caan BJ and Edwards S (1997) Hormone replacement therapy, reproductive history, and colon cancer: a multicenter, case–control study in the United States. *Cancer Causes Control* **8**: 146–158

Kuiper GGJM and Gustafsson J-A (1997) The novel estrogen receptor β subtype: potential role in the cell- and promoter-specific actions of estrogens and anti-estrogens. *FEBS* **410**: 87–90

La Vecchia C and Franceschi S (1991) Reproductive factors and colorectal cancer. *Cancer Causes Control* **2**: 193–200

Martinez ME, Grodstein F, Giovannucci E, Colditz GA, Speizer FE, Hennekens C, Rosner B, Willett WC and Stampfer MJ (1997) A prospective study of reproductive factors, oral contraceptive use, and risk of colorectal cancer. *Cancer Epidemiol Biomarkers Prev* **6**: 1–5

Negri E, La Vecchia C, Parazzini F, Savoldelli R, Gentile A, D'Avanzo B, Gramenzi A and Franceschi S (1989) Reproductive and menstrual factors and risk of colorectal cancer. *Cancer Res* **49**: 7158–7161

Negri E, Tzonou A, Beral V, Lagiou P, Trichopoulos D, Parazzini F, Franceschi S, Booth M and La Vecchia C (1999) Hormonal therapy for menopause and ovarian cancer in a collaborative re-analysis of European studies. *Int J Camcer* **80**: 848–851

Parkin DM, Whelan SL, Ferlay J, Raymond L and Young J (1997) *Cancer Incidence in Five Continents Vol. VII.* IARC Scientific Publication No 143.p International Agency for Research on Cancer: Lyon

Rannevik G, Jeppsson S, Johnell O, Bjerre B, Lurell-Borulf Y and Svanberg L (1995) A longitudinal study of the perimenopausal transition: altered profiles of steroid and pituitary hormones, SHBG and bone mineral density. *Maturitas* **21**: 103–113

Talamini R, Franceschi S, Dal Maso L, Negri E, Conti E, Filiberti R, Montella M, Nanni O and La Vecchia C (1998) The influence of reproductive and hormonal factors on the risk of colon and rectal cancer in women. *Eur J Cancer* **34**: 1070–1076

Troisi R, Schairer C, Chow W-H, Schatzkin A, Brinton LA and Fraumeni JF Jr (1997) Reproductive factors, oral contraceptive use, and risk of colorectal cancer. *Epidemiology* **8**: 75–79

© 2000 Cancer Research Campaign
